# Habitat-related differences in song structure and complexity in a songbird with a large repertoire

**DOI:** 10.1186/s12898-019-0255-7

**Published:** 2019-09-18

**Authors:** Krzysztof Deoniziak, Tomasz S. Osiejuk

**Affiliations:** 1 Department of Behavioural Ecology, Institute of Environmental Sciences, Faculty of Biology, Adam Mickiewicz University, Umultowska 89, 61-614 Poznań, Poland; 2 Laboratory of Insect Evolutionary Biology and Ecology, Institute of Biology, University of Białystok, Ciołkowskiego 1J, 15-245 Białystok, Poland

**Keywords:** Animal communication, Birdsong, Songbirds, *Turdus philomelos*, Urban ecology, Anthropogenic noise, Urbanisation

## Abstract

**Background:**

Urbanisation has been shown to influence many aspects of animal vocal communication. Much attention has been paid to anthropogenic noise, which is often described as one of the most challenging disturbances for urban dwellers. While a large body of literature describes associations between vocal behavior of avian populations and background noise level, most of these studies were conducted on species with relatively simple songs and small repertoire sizes. This study focuses on the song thrush, *Turdus philomelos*, a common Eurasian songbird with a complex singing style and large syllable repertoire. Our objective was to determine whether frequency, repertoire and temporal organisation of song parameters vary between birds inhabiting urban and adjacent forest habitats in which ambient noise levels differ.

**Results:**

Songs of urban males were found to be more complex than in conspecifics from natural forest populations. Urban dwellers possessed greater syllable repertoires and repeated syllable sequences more often. In addition, they used a smaller proportion of whistles and a higher proportion of twitter syllables when singing compared to the nonurban males. Moreover, we found significant differences in the minimum and peak frequency of the whistle syllable between studied populations.

**Conclusions:**

These findings may be an example of adaptation of acoustic communication in noisy urban environments, but we also discuss other possible explanations. We emphasize the need for further investigation into the relationships between birdsong and habitat characteristics, male quality, population density and ambient noise level in populations occupying urban and nonurban habitats.

## Background

Habitat characteristics can strongly affect animal vocalizations [1–3] due to various factors such as ground surface and vegetation type, microclimate conditions or noise from different biotic and abiotic sources [4, 5]. To improve signal transmission, animals adjust their acoustic communication to the environmental conditions of the occupied habitat. This has been observed in various taxa, including amphibians [6], birds [7, 8] and mammals [9, 10]. Such phenotypic adjustment may result from microevolutionary processes leading to local adaptation or phenotypic flexibility [11].

Urbanised landscapes are novel habitats when measured on an evolutionary scale. Since the industrial revolution they have expanded rapidly and currently cover approximately 3% of the global land surface [12], resulting in various environmental and ecological issues [13–15]. In recent years, the effect of anthropogenic noise has received great attention, and is often described as one of the most challenging disturbances in urban habitats [16]. It not only affects human health [17] but also shapes animal acoustic communication [18]. Studies report changes in vocalizations in various animal taxa due to exposure to anthropogenic noise [e.g., 19, 20] and show acoustic signal variation between populations of the same species occupying urban and nonurban habitats [e.g., 21, 22].

In recent years, much attention has been paid to the vocal behaviour of avian populations living in urban landscapes [16]. The majority of bird species communicate primarily via acoustic signals which play an important role during everyday activities such as mate attraction or territorial defence [23]. The ability for individuals to hear and be heard can become increasingly difficult under noisy conditions [18, 24]. Anthropogenic noise creates a new and demanding environment for efficient acoustic communication, interfering with the acoustic signals of birds by decreasing their signal-to-noise ratio [e.g., 25]. However, studies show that urban dwellers are able to adjust their song to an ambient noise level by modifying the spectral characteristics of their song [26–28], changing song duration and singing rate [29, 30], increasing signal amplitude [31] or shifting the timing of their singing [32–35], compared to individuals from non-urban populations.

Observing variation in avian acoustic signals in natural versus noisy environments demands careful consideration with respect to the mechanisms regarding signal response and functionality [36]. There is still an ongoing debate on the causes of the reported differences in song characteristics between habitats. An example of this is the widely described upward song frequency shift, demonstrated in various bird species as a response to anthropogenic noise. Apart from being a by-product of singing louder, a phenomenon known as the Lombard effect [18, 37, 38], recent research considers the possibility that this may be in part a result of measurement errors that could potentially lead to false positives [39–42]. Furthermore, different responses to anthropogenic and natural noise were observed in various species. In the Pacific wren (*Troglodytes pacificus)*, proximity to natural versus anthropogenic noise sources had significantly different effects on syllable length and song duration [43], while for the common chaffinch, *Fringilla coelebs*, there was only an increase in signal redundancy in noisy natural habitats [44, 45]. Reported changes in song characteristics can be due to the fact that noise is not the only factor present in urban habitats that influences acoustic communication [46]. Individuals can respond to variations in environmental conditions like habitat characteristics [8, 47–49] or population densities [50] and change their phenotypic state or activity [51] which may be reflected in their acoustic signalling.

The objective of this study was to examine the divergence in acoustic characteristics between urban areas and surrounding natural forests for a species with a complex song. It is less known how species with large repertoires and high syllable repetition cope with the acoustic pressures found in noisy urban environments. In more frequently studied species with relatively simple songs, the female’s decision on a suitable mate is not made based on the male’s repertoire, as this repertoire is used during demonstrations of territorial aggression (i.e. switches between song types or song type matching) [23]. However, in species with complex repertoires, repertoire size is driven by intersexual selection and functions as an indicator of male fitness [52, 53]. Therefore, noise can not only impair song detectability in complex singers, but also the perception of information it carries. Hence, the mechanisms to overcome the negative effect of noise could had evolved in different way in species with simple and complex repertoires due to the various functions of song characteristics.

**Fig. 1:**
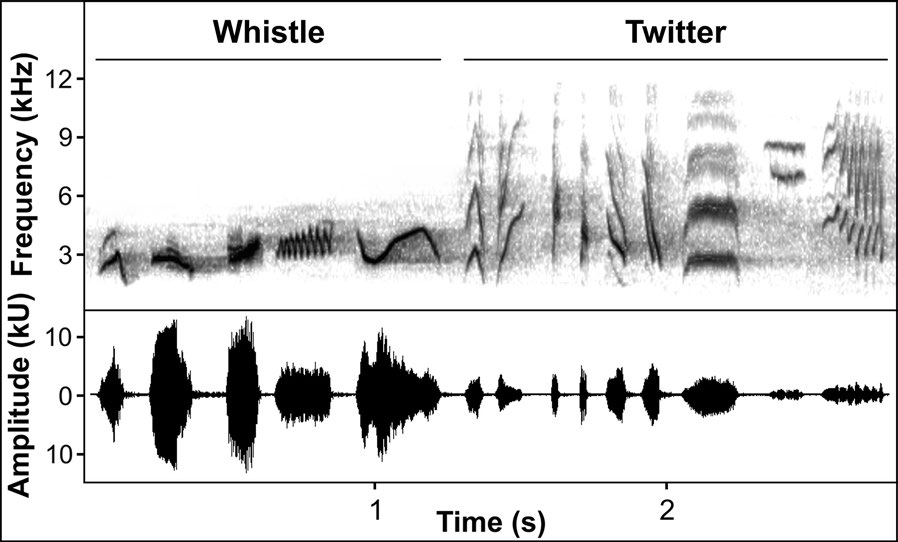
Spectrogram and waveform of typical song thrush syllables categorized as whistles and twitters

For this reason, for our model species, we chose the song thrush, *Turdus philomelos*, a Eurasian songbird inhabiting both forest and forest-like habitats within cities. Song thrushes belong to a group of bird species possessing complex songs and a large repertoire [54], with a characteristic, species-specific repetition of certain syllables during song production. Such syllables can be roughly categorized into two distinct types: low-frequency loud whistles and wide-band soft twitters (Fig. 1). Based on knowledge from other species it can be expected that acoustically different syllable types are used to address various receivers and/or receivers within distinct ranges [23, 55]. Both types of syllables resemble those in the repertoire of the closely related common blackbird *Turdus merula*, whose song has been studied more intensively [i.e., 55–57]. Therefore, it is possible that the functions of whistle and twitter syllables are similar for both species, but this needs further testing. Loud and simple whistle syllables are adapted to long-range communication and can be heard far beyond male territory borders. On the other hand, twitter syllables are greatly limited by vegetation [4, 58] and seem to be better suited for short-range communication. Whistles and twitters are both repeated in sequences, increasing the redundancy of the song and therefore making it more detectable [5].

For the present study, we focused on song characteristics describing the frequency, repertoire and temporal organization of song output. We predicted that males would spectrally adjust their song to avoid masking from noise when singing in urban habitats, as was observed in the blackbird [59] and other songbirds [e.g., 60, 61]. For this reason, we expected that song thrushes would show a habitat-related frequency shift of their song in the noisy environment. Furthermore, we also predicted that the noisy urban environment could have different effects on the acoustically distinctive whistles and twitters. These differences might be related to a divergence in frequency shift, as low-pitched whistles may be masked more, by urban noise, than the wide-band twitters that also have energy at relatively higher frequencies. This may also concern the proportions at which both types of syllables are produced in different environments. In general, song thrushes apply a pattern of syllable production with some of them having clear repetition in a series. If the repetition of syllables improves signal transmission, we expected to observe increased syllable redundancy in males singing in noisy urban environments. This may be observed to a greater extent in twitter syllables, since they are of a considerably lower sound pressure level and shorter transmission range that whistles. Finally, we wanted to take a closer look at the variation in repertoire size between males from urban and natural forest populations. Although a direct effect of noise on repertoire size seems to be unlikely, a potential difference may be caused by heterogeneity in habitat complexity [8] or other anthropogenic disturbances present in urban environments [62].

## Methods

### Study area

The study was conducted in the Wielkopolska region in Western Poland. Male song thrushes were recorded in urban forests within the city of Poznań (N52°25.191′, E16°55.795′; 23 males recorded) and in natural forests surrounding the city to the north (35 males recorded). Study site map with recording locations is shown in Additional file 1: Figure S1. Urban sites consisted of two large woodland patches with a dominance of temperate and mixed coniferous forest and were surrounded by a high density of urban development (housing, industry, major roads). Recorded song thrushes were singing within a distance of 3.5 km to 6.0 km from the city centre, avoiding forest edges. Natural forest sites consisted of two large temperate and mixed coniferous forests surrounded by farmland. Only males singing from the centre of forest habitat fragments were recorded. We avoided areas with recent or ongoing logging conducted by the State Forests. Considering the matrix of suitable habitat types present at both urban and non-urban sites, it is likely that potential differences in male song parameters would originate from sources such as individual variation (e.g., quality, social environment) or micro-scale differences (e.g., micro-habitat, noise), rather than macro-scale differences (belonging to areas of different dialects).

### Song recording

Song thrush songs were recorded in the first four hours after sunrise during four breeding seasons (2012–2015) from March until July. To exclude the possibility of recording a particular bird more than once, each male was recorded in a different location, and that location was not visited again in the same year or following seasons. We used a Marantz PMD670 recorder (Marantz Professional, Kanagawa, Japan) and a Telinga Pro 6 microphone mounted on a Telinga Universal parabola (Telinga Microphones, Uppsala, Sweden). Recordings were saved as mono-linear 48 kHz/16 bit PCM WAV files. Noise level measurements were conducted with a CHY 650 digital sound level meter (range 35–130 dB SPL re 20 μPa; frequency weighting: A; fast response; ANSI S1.4, Class II). Ten noise level measurements were obtained after each recording and averaged for later data analysis. Recordings were only made during days with no rain and with low wind speeds (< 5 m/s measured with a Voltcraft PL-130 anemometer; Conrad Electronics, Hirschau, Germany).

### Acoustic analysis

We used Raven Pro 1.5 Beta v. 23 (Cornell Lab of Ornithology, Ithaca, USA) to measure the repertoire and temporal characteristics for 1000 consecutive syllables of continuous song from each male (for a syllable examples, see Fig. 1). Measurements were taken from spectrograms with the following parameters: DFT size: 256, frequency resolution: 188 Hz, window type: hamming, overlap: 50%. All males were blind-coded so that the authors were unaware of their origin during acoustic analysis. We incorporated syllable and repertoire classification methodology from a previous study on the closely related common blackbird, as its song structure resembles that of the song thrush [56]. A syllable was defined as a single element without sound-free pauses that were longer than 0.015 s. This allowed us to obtain syllable duration (s) and inter-syllable intervals (s) which we used to measure syllable rate (number of syllables produced per minute). Afterwards, repertoire sizes were determined by counting the number of different syllables within the sample of 1000 consecutive syllables. Syllables were classified on the basis of their overall appearance, regarding the frequency and temporal characteristics visible on the spectrogram. Each syllable was also assigned to one of two fundamental categories: whistle or twitter (Fig. 1). Whistles were almost purely tonal, loud syllables with a lower frequency. Twitters had a broader frequency band with more harmonics and were generally of lower amplitude. Repertoire song characteristics were presented as repertoire size (number of different syllable types within the 1000 syllables measured for each male), repertoire of whistles (number of different whistle types within the overall repertoire), and repertoire of twitters (number of different twitter types within the overall repertoire). We also counted the difference in whistle and twitter fractions amongst the 1000 syllables recorded per male (twitter fraction).

Song thrush males sing with a characteristic repetition of syllables, where different syllable types could be produced with variable temporal patterns and delivered with eventual or immediate variety [23]. The more repetitions of the same syllable type, the smaller the overall repertoire size measured within a fixed number of syllables (1000) and the higher the redundancy index. The redundancy index is defined with the following formula:

$$Redundancy\; index = \frac{sum \;of \;transitions \;between\; unique \;syllable\; types}{sum \;of \;unique\; syllable\; types - 1}$$

The redundancy index is 1.0 when a bird is singing the same syllable type all the time and reaches 0 if a bird constantly switches between different syllable types. Song thrushes in our dataset were found to produce repeated syllables between two and nineteen times in a series. Therefore, we used a linearity index to measure the sequential complexity of the song [63]. A linearity index was calculated using the following formula:

$$Linearity \;index = \frac{number \;of \;unique\; syllable \;types}{transitions\; between\; different\; syllables\; types + 1}$$

The index is 1.0 when the syllable sequence in the song is always identical, and it will approach 0 when the syllable sequence is completely random. It is worth noting that although both indices refer to syllable repetition within a sample, they describe different aspects of song complexity. For example, a male with only two different syllables in his repertoire may sing with immediate variety (ABABAB…) or eventual variety (AAABBB…), which dramatically affects the linearity index, while the redundancy index remains the same.

Measurements of birdsong frequency characteristics that are obtained via positioning the computer cursor over the spectrogram are prone to errors and can lead to observer-expectancy biases [e.g. 39, 41]. Therefore, we used the automatic parameter measurements function in Avisoft SASlab Pro v. 5.2.12 (Avisoft Bioacoustics, Berlin, Germany) to measure the minimum frequency (Hz) and peak frequency (Hz) for 1000 syllables from each male. All of the measurements were taken with the following parameters: FFT length: 1024, frame size: 100%, window type: hamming, temporal overlap: 75. We applied a high-pass filter (cut off-frequency: 800 Hz) and set an amplitude threshold of − 12 dB below the peak in a power spectrum. We chose a − 12 dB measurement threshold for the power spectra after preliminary analysis of recordings with the lowest signal-to-noise ratio and comparing them with those with higher signal-to-noise ratios. After conducting automatic measurements, we visually inspected the whole dataset to detect errors that could result from overlapping with noises caused by natural and anthropogenic sources and excluded them from further analysis.

### Statistical analysis

Prior to analysis, we tested the variables for normality using a Kolmogorov–Smirnov one-sample test. Each tested variable except for the inter-syllable intervals had a normal distribution. We then used Student's *t*-test or Mann–Whitney *U*-test, respectively to variable distribution, to examine whether parameters describing the syllable frequency, repertoire and temporal song organization varied significantly between urban and natural forest populations (Table 1). Next, we applied general linear models (GLM) for seven variables that differed significantly between studied populations (Table 2). The predictor variables and covariates used in the GLM were as follows: day in a season, hour after sunrise, habitat type (urban = 1; forest = 2), level of ambient noise present in the recorded territory, and the presence of other singing males within the hearing range of the recorded territory (if the recorded male was singing alone = 0; presence of other singing males in the background = 1). An information-theoretic approach was used to compare candidate models on the basis of the Akaike’s Information Criterion (AIC_C_) corrected for small sample sizes. Models were ranked with Δ AIC_C_, which is the difference between the best (lowest) AIC_C_ value and the AIC_C_ value for every other model. Values from the best fitted models (ΔAIC_C_ < 2) were converted to two measures that could be used to assess the relative strengths of models [64]. The Akaike weight (*w*_*i*_) was used to provide normalised relative model likelihoods, with higher values indicating the model with the best predictor set. Evidence ratios (ERs) allowed for the comparison of models, with values from a particular model being compared to the best fitted model available [65]. All of the means are presented with their accompanying SD, unless otherwise indicated. All of the statistical analyses were two-tailed and were performed using IBM SPSS Statistics v. 24 (IBM Corp, Chicago, IL, USA). All figures were created with the IBM SPSS Statistics v. 24 and CorelDRAW X5 (Corel Corporation, Ottawa, Canada) and are presented as untransformed data.

**Table 1:** Differences in song thrush song characteristics between studied populations

	Variable	Urban 95% CI	Forest 95% CI	t/*Z*	p
A	Whistle minimum frequency	2465.4–2538.9	2312.0–2421.7	3.778	*< 0.001*
	Whistle peak frequency	2978.0–3112.5	2803.6–2945.9	3.369	*0.001*
	Twitter minimum frequency	3939.2–4254.6	3823.2–4051.1	1.725	0.090
	Twitter peak frequency	5149.8–5459.2	5008.9–5262.4	1.725	0.090
B	Syllable repertoire	343.58–422.85	276.05–335.72	3.246	*0.002*
	Whistle repertoire	117.78–146.48	97.89–118.96	2.787	*0.007*
	Twitter repertoire	216.14–286.04	172.83–222.08	2.649	*0.010*
	Twitter fraction	433.06–543.64	366.31–462.49	2.035	*0.047*
C	Syllable duration	0.10–0.11	0.10–0.11	− 0.337	0.737
	Inter-syllable intervals	0.33–0.42	0.36–0.45	− 0.715	0.474
	Syllable rate	122.19–145.07	116.64–136.93	0.898	0.373
	Redundancy index	0.20–0.24	0.22–0.26	− 1.138	0.260
	Linearity index	0.44–0.54	0.36–0.44	2.811	*0.007*

Characteristics of song parameters describing (A) frequency, (B) repertoire and (C) temporal organization. Statistics: Mann–Whitney’s *U*-test (inter-syllable intervals) and Student's *t*-tests (all other variables). Significant differences are in italics

**Table 2:** General linear models assessing variation in song thrush song characteristics that differed significantly between studied habitats

Model	AIC_C_	ΔAIC_C_	*w*_*i*_	ER
Whistle minimum frequency				
HABITAT	740.66	0.00	0.39	
HABITAT + MALES	742.05	1.40	0.19	2.01
HABITAT + HOUR	742.10	1.44	0.19	2.05
Whistle peak frequency				
HABITAT	776.56	0.00	0.34	
HABITAT + HOUR	777.53	0.97	0.21	1.62
HABITAT + DAY	777.79	1.23	0.18	1.85
HABITAT + DAY + HOUR	778.32	1.76	0.14	2.41
HABITAT + NOISE	778.40	1.84	0.13	2.51
Syllable repertoire				
NOISE + DAY	684.54	0.00	0.32	
NOISE	685.04	0.50	0.25	1.28
NOISE + DAY + HOUR	685.72	1.18	0.18	1.80
NOISE + DAY + MALES	686.24	1.70	0.14	2.34
NOISE + MALES	686.43	1.89	0.12	2.58
Whistle repertoire				
NOISE	567.04	0.00	0.30	
NOISE + MALES	567.17	0.13	0.29	1.07
NOISE + HOUR	568.48	1.43	0.15	2.05
NOISE + DAY + HOUR	568.74	1.69	0.13	2.33
NOISE + DAY	568.76	1.72	0.13	2.36
Twitter repertoire				
NOISE + DAY	664.54	0.00	0.41	
NOISE + DAY + HOUR	666.03	1.49	0.19	2.11
Twitter fraction				
NOISE + DAY	736.76	0.00	0.27	
NOISE	737.13	0.37	0.22	1.20
HABITAT + DAY	737.42	0.66	0.19	1.39
DAY	737.58	0.83	0.18	1.51
HABITAT	738.10	1.34	0.14	1.96
Linearity index				
NOISE	− 85.43	0.00	0.36	
NOISE + DAY	− 84.26	1.17	0.20	1.80
NOISE + HOUR	− 83.78	1.65	0.16	2.28
NOISE + MALES	− 83.73	1.70	0.15	2.34

Models with the highest probability (ΔAIC_C_ < 2) are shown. The Akaike weight (*w*_*i*_) and evidence ratio (ER) were calculated on the basis of Akaike’s Information Criterion corrected for small sample size (see “Methods” for details). Predictor codes: DAY, day of season; HOUR, hour after sunrise; NOISE, background noise level; HABITAT, habitat type; MALES, other singing males in hearing range during recording

## Results

**Fig. 2:**
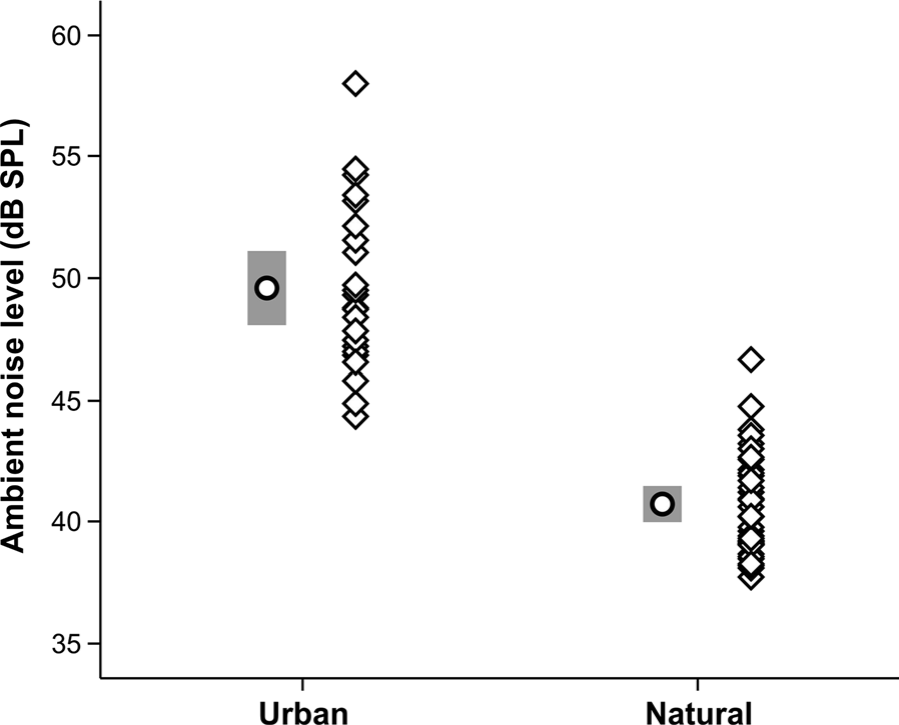
Differences in background noise level (dB SPL) within and between studied habitat types. Each diamond represents a single recorded male. Box plot shows means and 95% CI

Ambient noise levels differed significantly between urban (49.6 ± 3.45 dB SPL, n = 23) and forest (40.7 ± 2.14 dB SPL, n = 35) sites (Student’s *t* test, t = 12.10, p < 0.001, n = 58; Fig. 2). Other singing males were present in the hearing range during the time of recording in 6 out of 23 cases in the urban site (26.1%) and in 9 out of 35 cases in the non-urban site (25.7%). There were no large differences in the time and date of the recordings from urban vs. nonurban habitats. Correlation matrices of song thrush song characteristics are shown in Additional file 2: Table S1, Table S2, Table S3.

### Syllable frequency

**Fig. 3:**
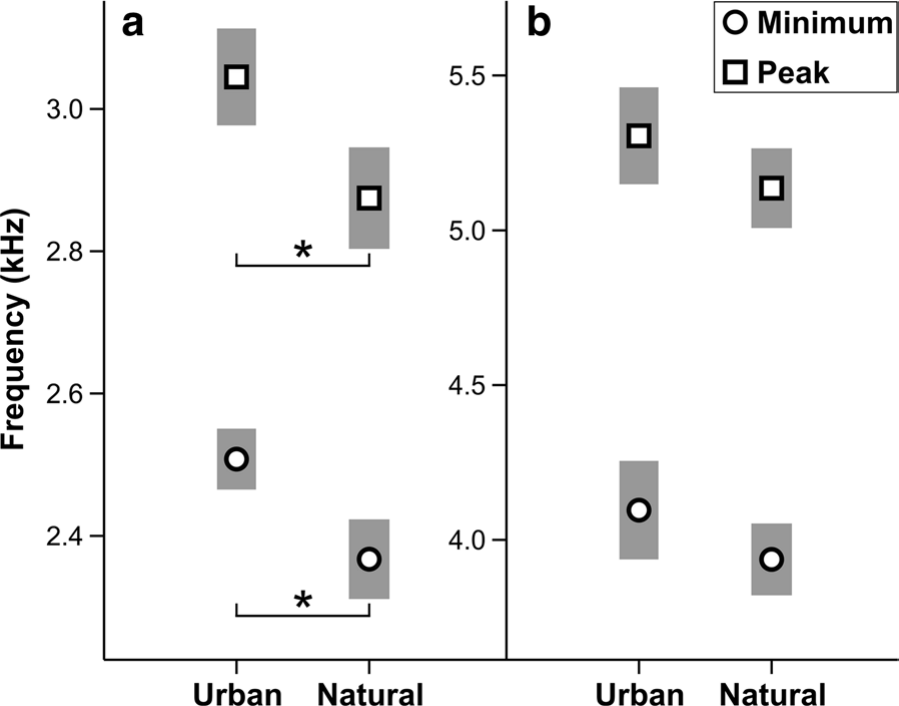
Differences in minimum and peak frequency of whistle (**a**) and twitter (**b**) syllables between studied habitats (means and 95% CI; *Student's *t*-test p-value < 0.05)

The song of urban males was characterized by a higher frequency of whistle syllables when compared to nonurban males (Table 1, Fig. 3). The best fitting GLM (ΔAIC_C_ < 2) included three possible model combinations for both variables (Table 2), where each model showed a significant effect of habitat type on the minimum and peak frequency of whistle syllables (Additional file 2: Table S4). Relationship between ambient noise level and whistle syllable frequency is shown in Additional file 1: Figure S2, Figure S3 and Additional file 2: Table S5. We found no significant differences in the twitter syllable minimum and peak frequency between the studied populations (Table 1).

### Repertoire size and use of whistle and twitter syllables

**Fig. 4:**
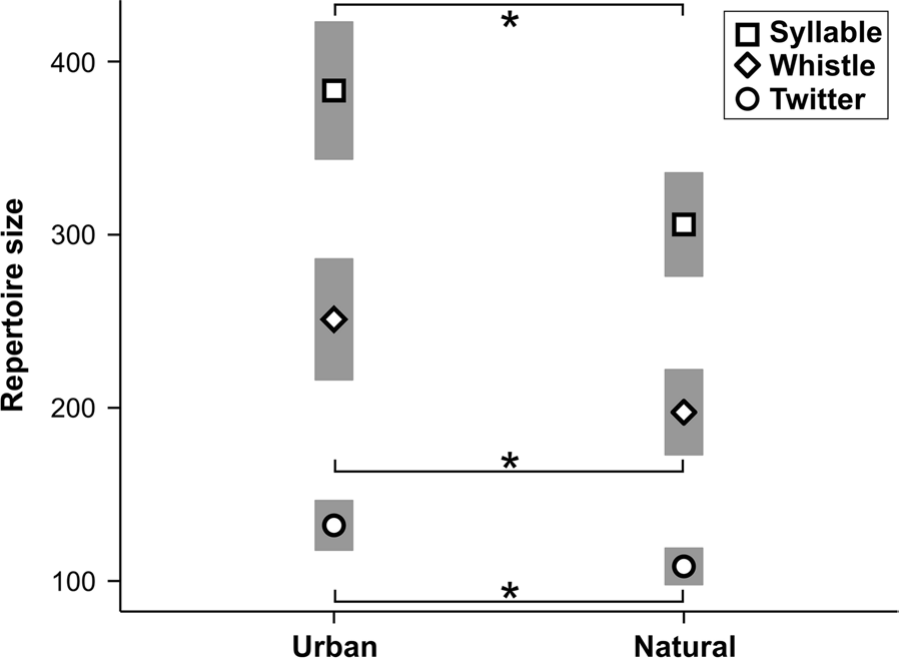
Differences in syllable, whistle and twitter repertoire within 1000 subsequent syllables of continuous song between studied habitats (means and 95% CI; *Student's *t*-test p-value < 0.05)

**Fig. 5:**
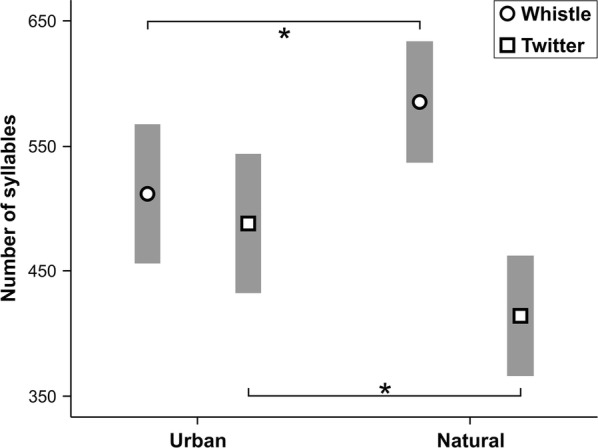
Differences in whistle and twitter fractions within 1000 subsequent syllables of continuous song between studied habitats (means and 95% CI; *Student's *t*-test p-value < 0.05)

Urban song thrushes had significantly larger overall syllable repertoires, as well as larger whistle and twitter repertoires, than their conspecifics from forest habitats (Fig. 4, Table 1). A positive correlation was found between syllable (r = 0.465, p < 0.001, n = 58; Additional file 1: Figure S4), whistle (r = 0.400, p = 0.002, n = 58; Additional file 1: Figure S5) and twitter (r = 0.393, p = 0.002, n = 58; Additional file 1: Figure S6) repertoires and the ambient noise level. Relationship between repertoire characteristics and ambient noise level is shown in Additional file 2: Table S5. Males produced significantly more twitters in urban populations (Fig. 5, Table 1). The best fitting GLM (ΔAIC_C_ < 2) for syllable repertoire and whistle repertoire included five possible model combinations for both syllable and whistle repertoire (Table 2), showing a significant effect of ambient noise level on both variables (Additional file 2: Table S4). A significant effect of ambient noise level, as well as the day in the season, was shown by the two best fitting GLMs. Here, the twitter repertoire decreased as the season advanced (Table 2, Additional file 2: Table S4). There were five best fitting GLMs that were shown for twitter fraction, with a significant effect of ambient noise level, day in season and habitat type for this variable (Table 2, Additional file 2: Table S4; Additional file 1: Figure S7). Estimates of ambient noise level revealed that syllable, whistle and twitter repertoires, as well as twitter fraction, were significantly higher in urban males (Additional file 2: Table S4).

### Temporal song organization

**Fig. 6:**
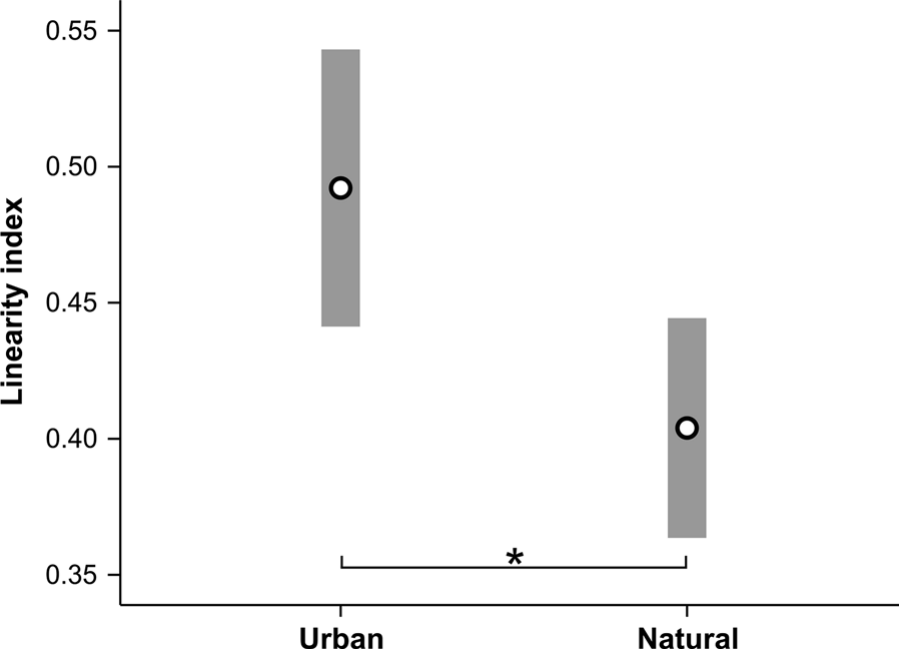
Differences in linearity index between studied habitats (means and 95% CI; *Student's *t*-test p-value < 0.05)

Males were shown to slowly decrease syllable production rate with seasonal progression (r = − 0.268, p = 0.042, n = 58). The redundancy and linearity indices describe different aspects of a songs temporal organization and were not significantly correlated (r = 0.200, p = 0.130, n = 58). The redundancy index did not differ significantly between urban and nonurban forest habitats (Table 1). However, the linearity index varied significantly between studied populations and was higher for urban males (Fig. 6, Table 1). The best fitting GLM (ΔAIC_C_ < 2) included four possible model combinations, each showing a significant effect of ambient noise level on the linearity index (Additional file 1: Figure S8; Additional file 2: Table S4). As predicted, song thrushes repeated syllable types more often before switching to another type while singing in noisy urban habitats (Additional file 2: Table S4). The linearity index was also highly correlated with repertoire size (r = 0.969, p < 0.001, n = 58) and twitter/whistle ratio (r = − 0.553, p < 0.001, n = 58). Therefore, in urban habitats, song thrushes repeated syllable sequences more often whilst increasing the proportion of twitter elements in their songs.

## Discussion

We have provided the first evidence of song divergence between urban and nonurban song thrushes, a species with complex song and a large syllable repertoire. As predicted, we observed an upward shift of the minimum and peak frequencies in urban males. A shift in syllable frequency might be an adaptation to reduce masking from noise as suggested in several studies [e.g., 26–28]. However, differences in song frequency were only shown in whistle syllables. Frequency of twitter syllables tended to be higher in the city, but this was not significant (p = 0.090). It seems likely that the shift in the low-pitched whistles may be because they are more exposed to masking by urban noise than the wide-band twitters. Although, a study on the closely related blackbird found that both whistle and twitter syllables had higher peak frequencies in urban males when compared to forest birds [48]. Our findings may also be caused by amplitude adjustment in urban song thrushes in response to anthropogenic noise, known as the Lombard effect. Studies have shown that the frequency and amplitude of acoustic signals are correlated and an increase in signal amplitude causes a passive response in its frequency (reviewed in [31]). This issue should be addressed in further studies since our work did not cover amplitude measurements.

Divergence in acoustically different syllables found between urban and forest habitats suggests an adaptation to communication in noisy urban environments. Here, twitter syllable redundancy was observed in two ways: by its larger fraction within the analysed samples and by the increased repetition in sequences. By making twitters more repetitive, song thrushes could compensate for masking of their song by noise and significantly improve their detectability in noisy urban environments [5]. In addition, twitters are characterized by their lower amplitude and so are more likely to be subject to attenuation and degradation than whistles [4, 58]. Such increased signal redundancy has been previously reported in king penguins *Aptenodytes patagonicus* [66] and chaffinches [44] living in noisy natural environments as well as in amphibians [67], birds [29] and mammals [68] exposed to anthropogenic noise. Alternatively, since urban noise and whistle syllables coincide in the lower frequencies, use of wide-band twitters may be an adaptation to reduce song masking from noise. However, a recent study found that blackbirds living next to a large airport were more likely to sing without or with a shorter twitter part than birds from a control population [69]. This change was explained by the fact that the twitter part of the blackbird song was almost fully masked by aircraft noise, which was not observed in our study under noisy urban conditions.

It remains unclear whether increased levels of ambient noise would induce such changes in birdsong. Previous studies on the common blackbird may explain the increased proportion of twitter syllables observed in urban population of song thrushes. Urban blackbirds were found to produce songs with larger twitter proportions, which was also positively correlated with territory densities [48]. If twitter syllables are adapted for short-range communication [55] we may expect that birds living in higher densities would use them more frequently due to increased interactions between individuals. Here, twitter syllables may also possess a function similar to that of “soft songs”, which are used to avoid eavesdropping or signal aggression [70, 71]. On the other hand, blackbirds from urban population were also found to sing with higher frequencies [48, 72]. Both studies suggested that singing with higher frequencies can be related to increased arousal levels of territorial males living in higher densities [73, 74]. Song thrush density was not assessed during our study. We monitored vocal activity of males in the background during song recordings, but observed no effect of their absence/presence on the analysed song characteristics. This may be due to the fact that it is difficult to estimate the population density of song thrushes [75]. Nonetheless, studies report that living in higher densities involves more intense social interaction with neighbours, which can be responsible for the increased aggression, arousal [76, 77] and stress [78, 79] observed in urban dwellers. This seems to have a direct effect on acoustic signalling in birds.

Differences found in the repertoire size between the studied habitats may also be side effects of urbanization. For example, a high abundance of food can contribute to song complexity, since it decreases the probability of nutritional stress during the juvenile period and allows proper development of the high vocal centre in birds [80, 81]. Food abundance in urban ecosystems is not only provided by feeders and waste food [76, 82], but also by the earlier plant phenology and invertebrate development [83–85] caused by the “urban heath island” [86], as well as the extended foraging time provided by artificial light [87, 88]. Moreover, the success of urban dwellers may be caused by increased brain size, which helps birds respond to novel conditions [89–91]. This can provide extended memory, allowing open-ended learning or re-expression of syllables learned earlier in their lifetime [92–94], as was suggested for repertoire plasticity observed in the clay-coloured thrush *Turdus grayi* [95]. Phenotypic quality and resource holding potential of individuals, as well as habitat complexity and quality, were also shown to contribute to increased song complexity in birds [8, 96–98]. Finally, if urban habitats are truly more attractive for song thrushes, we should expect higher individual density and intense competition over available resources [99, 100] which can be reflected in various attributes of communication complexity [101] as was observed in primates [102], sciurid rodents [103] and birds [104, 105].

## Conclusion

In conclusion, in this first multidimensional analysis of song thrush song, we show habitat-related variation in the structure and temporal organization of song characteristics. Urban dwellers appeared to be more complex singers, based on either larger syllable, whistle and twitter repertoires or increased syllable sequence repetition. Moreover, change in frequency of syllables produced by urban males, which, together with discrepancies in the proportions of whistle and twitter syllables between studied populations, is a potential example of adaptations to acoustic communication in noisy urban environments. However, since birdsong is a complex signal, it is not easy to specify a particular factor, or a combination of several factors, from which those differences originate. Accumulating evidence suggests that at least some of the observed changes in song thrush song characteristics may be a side effect of urbanisation, rather than a direct response to a greater level of anthropogenic noise. Many of those differences seem to be indirectly connected to population density, but that was not assessed during our study. Further investigation is needed to define and confirm the relationships between ambient noise levels, population densities, male quality and several characteristics of birdsong.

## Supplementary information

**Additional file 1: Figure S1.** Map of the study areas. Red circles represent locations where song thrushes were recorded. Map generated from OpenStreetMap open data, licensed under the Open Data Commons Open Database License by the OpenStreetMap Foundation (https://www.openstreetmap.org/). **Figure S2.** Relationship between the whistle peak frequency and ambient noise level in studied habitats. **Figure S3.** Relationship between the whistle minimum frequency and ambient noise level in studied habitats. **Figure S4.** Relationship between syllable repertoire size (number of unique syllable types within 1000 subsequent syllables of continuous song) and ambient noise level in studied habitats. **Figure S5.** Relationship between whistle repertoire size (number of unique whistle syllable types within 1000 subsequent syllables of continuous song) and ambient noise level in studied habitats. **Figure S6.** Relationship between twitter repertoire size (number of unique twitter syllable types within 1000 subsequent syllables of continuous song) and ambient noise level in studied habitats. **Figure S7.** Relationship between twitter fraction (number of twitter syllables within 1000 subsequent syllables of continuous song) and ambient noise level in studied habitats. **Figure S8.** Relationship between linearity index and ambient noise level in studied habitats.

**Additional file 2: Table S1.** Correlation matrices of song parameters from song thrush males recorded in urban forests and natural forests. Significant values are indicated in bold. **Table S2**. Correlation matrices of song parameters from song thrush males recorded in urban forests. Significant values are indicated in bold. **Table S3**. Correlation matrices of song parameters from song thrush males recorded in natural forests. Significant values are indicated in bold. **Table S4**. Results of the best fitting general linear models explaining the variation in song characteristics that differed between the studied habitats. **Table S5**. Results of the linear regression models explaining the relationship between ambient noise levels on song thrush song characteristics. Models show results for urban habitat, forest habitat and the data combined.

## Data Availability

The datasets used and/or analysed during the current study are available from the corresponding author on reasonable request (Additional files 1, 2).

## Abbreviations

GLM: general linear models
AIC_C_: Akaike’s Information Criterion corrected for small sample sizes
*w*_*i*_: akaike weight
ER: evidence ratio
DAY: day of season
HOUR: hour after sunrise
NOISE: background noise level
HABITAT: habitat type
MALES: other singing males in hearing range during recording
SPL: sound pressure levels
dB: decibel
km: kilometer
μPa: micropascal
kHz: kilohertz
s: second
fig: figure
