# Supplementary material for: Habitat-related differences in song structure and complexity in a songbird with a large repertoire
Source: BMC Ecol. 2019 Sep 18;19:40. doi: 10.1186/s12898-019-0255-7 (PMC6749692; doi:10.1186/s12898-019-0255-7)
Supplement: Supplementary file 1 — Additional file 1: Figure S1. Map of the study areas. Red circles represent locations where song thrushes were recorded. Map generated from OpenStreetMap open data, licensed under the Open Data Commons Open Database License by the OpenStreetMap Foundation (https://www.openstreetmap.org/). Figure S2. Relationship between the whistle peak frequency and ambient noise level in studied habitats. Figure S3. Relationship between the whistle minimum frequency and ambient noise level in studied habitats. Figure S4. Relationship between syllable repertoire size (number of unique syllable types within 1000 subsequent syllables of continuous song) and ambient noise level in studied habitats. Figure S5. Relationship between whistle repertoire size (number of unique whistle syllable types within 1000 subsequent syllables of continuous song) and ambient noise level in studied habitats. Figure S6. Relationship between twitter repertoire size (number of unique twitter syllable types within 1000 subsequent syllables of continuous song) and ambient noise level in studied habitats. Figure S7. Relationship between twitter fraction (number of twitter syllables within 1000 subsequent syllables of continuous song) and ambient noise level in studied habitats. Figure S8. Relationship between linearity index and ambient noise level in studied habitats. [file 12898_2019_255_MOESM1_ESM.docx]

**Additional file 1: Figures S1-S8**

**BMC Ecology**

**DOI: 10.1186/s12898-019-0255-7**

**Title:** Habitat-related differences in song structure and complexity in a songbird with a large repertoire

**Authors:** Krzysztof Deoniziak ^1, 2^, Tomasz S. Osiejuk ^1^

**Affiliation:**

^1^ Department of Behavioural Ecology, Institute of Environmental Sciences, Faculty of Biology,
Adam Mickiewicz University, Umultowska 89, 61-614 Poznań, Poland

^2^ Laboratory of Insect Evolutionary Biology and Ecology, Institute of Biology, University of Bialystok, Ciołkowskiego 1J, 15-245 Białystok, Poland

**Corresponding author email:** [krzysztofdeo@gmail.com](mailto:krzysztofdeo@gmail.com)

**
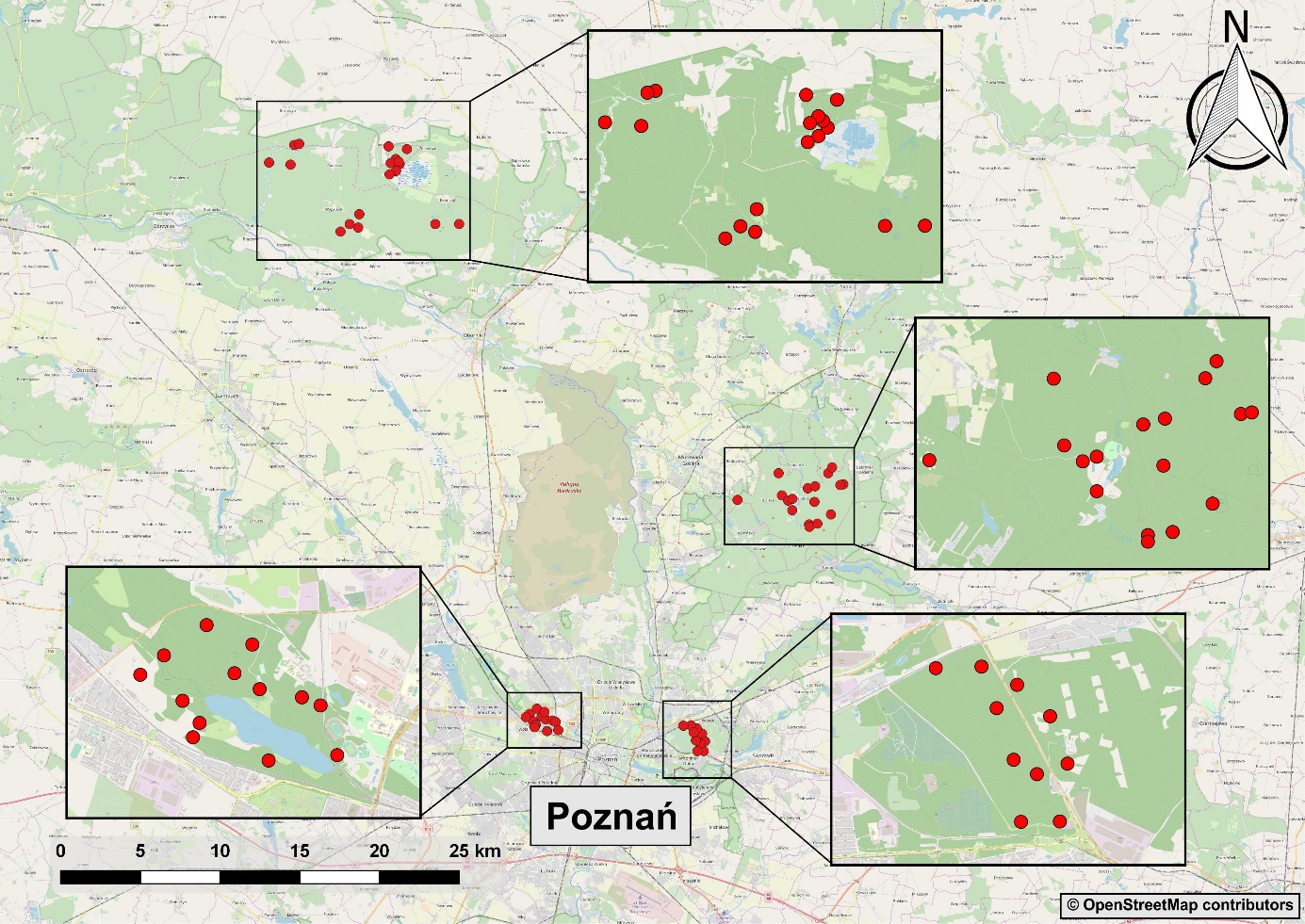
**

**Figure S1:** Map of the study areas. Red circles represent locations where song thrushes were recorded. Map generated from OpenStreetMap open data, licensed under the Open Data Commons Open Database License by the OpenStreetMap Foundation (https://www.openstreetmap.org/)

**
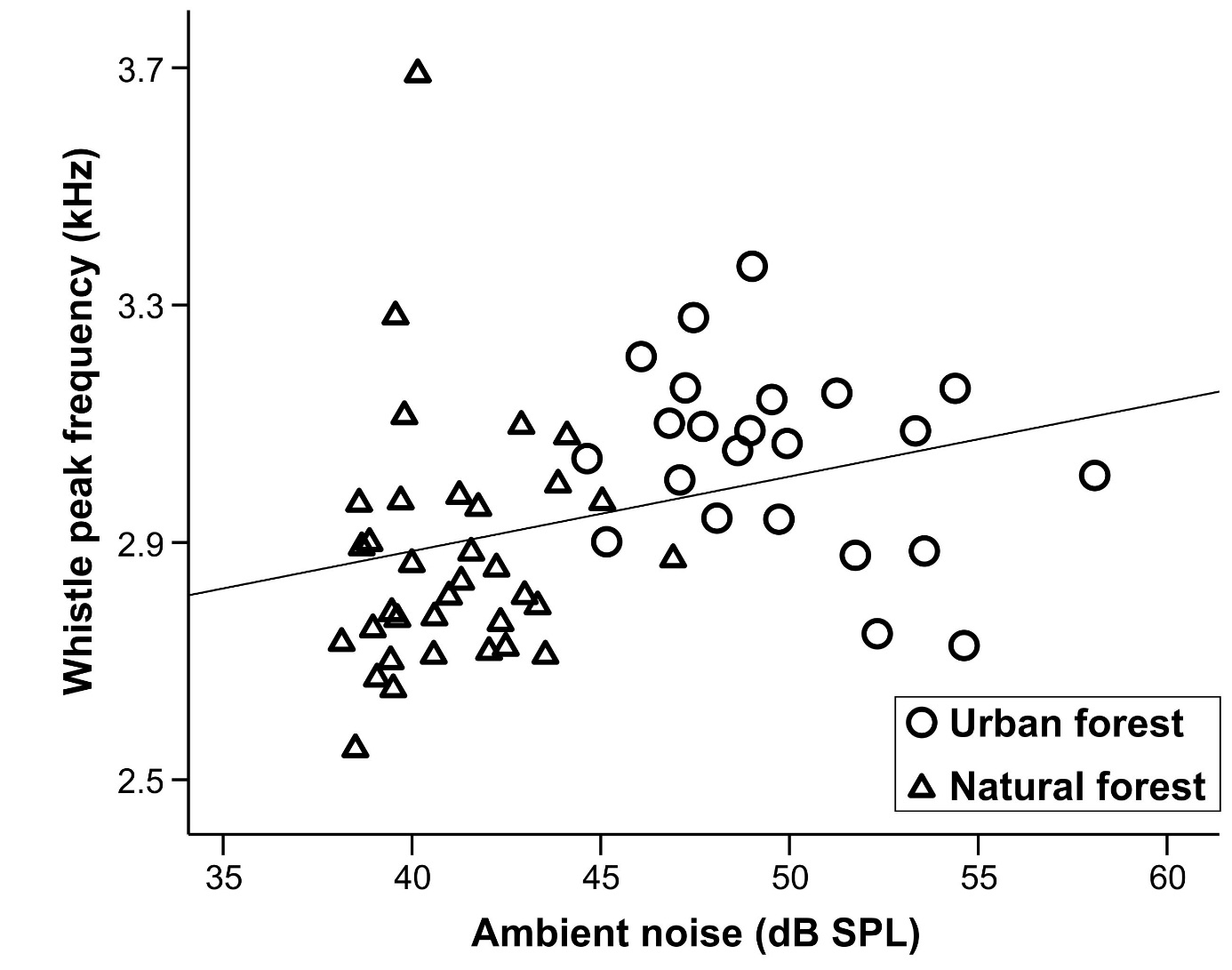
**

**Figure S2:** Relationship between the whistle peak frequency and ambient noise level in studied habitats.

**
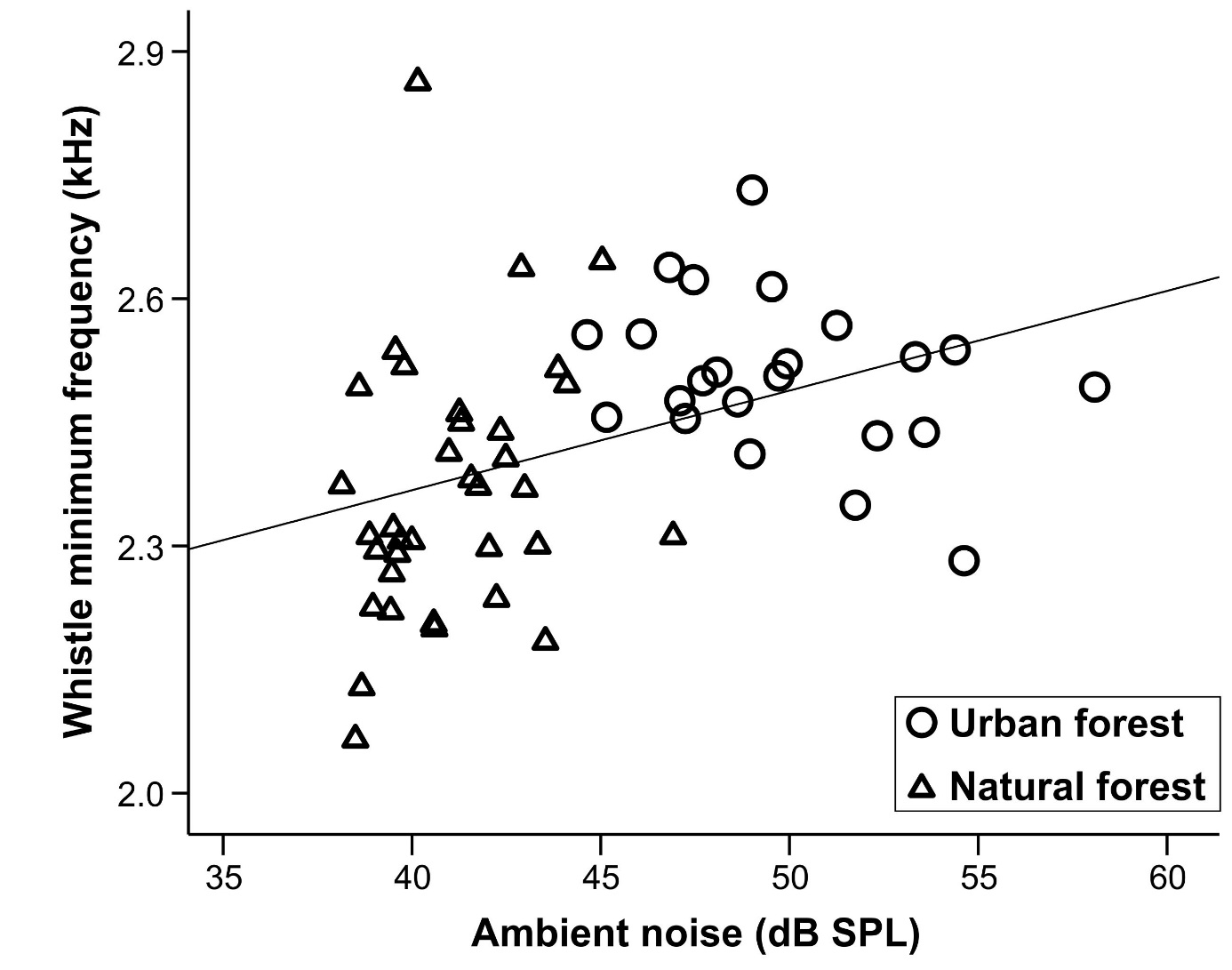
**

**Figure S3:** Relationship between the whistle minimum frequency and ambient noise level in studied habitats.

**
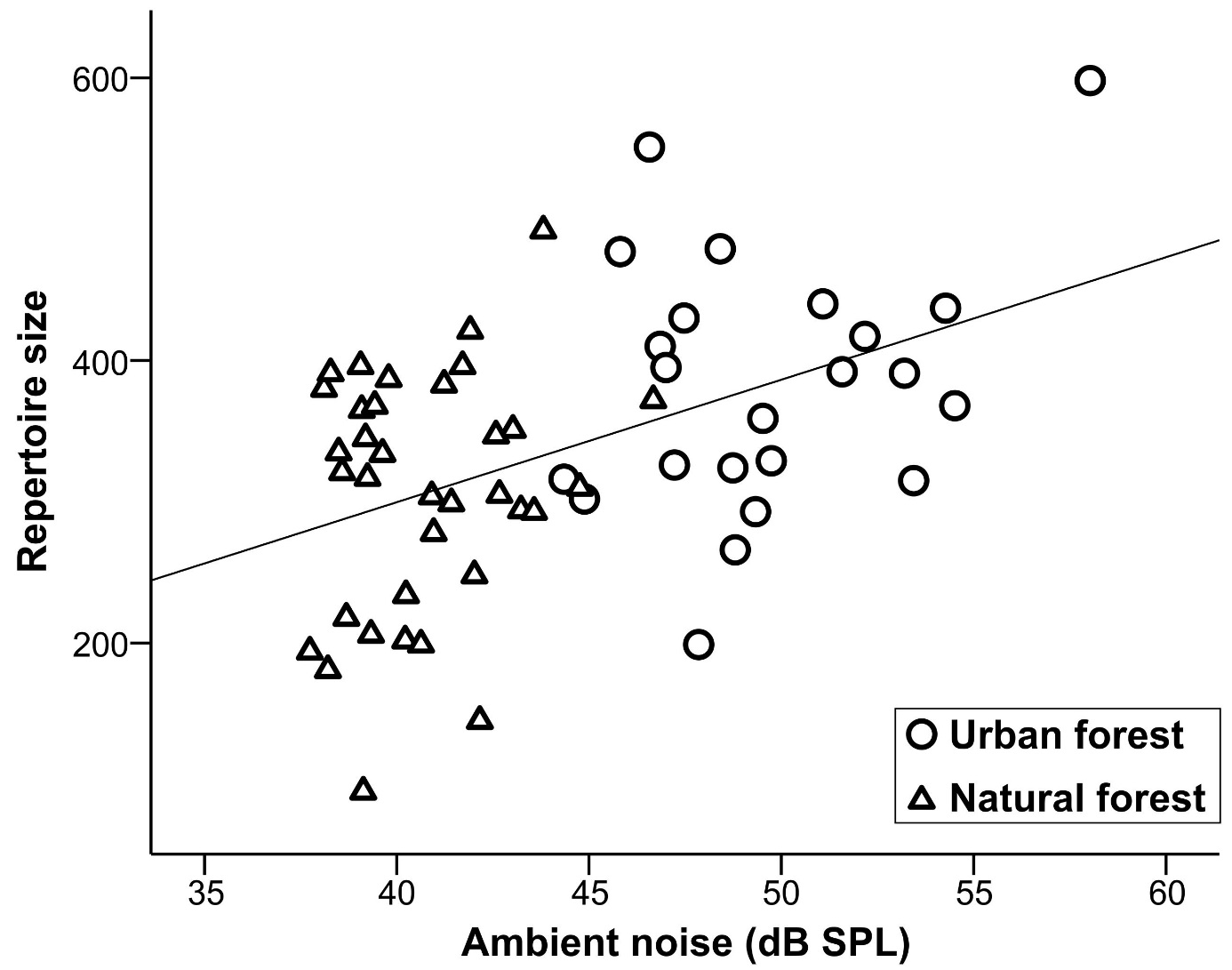
**

**Figure S4:** Relationship between syllable repertoire size (number of unique syllable types within 1000 subsequent syllables of continuous song) and ambient noise level in studied habitats.

**
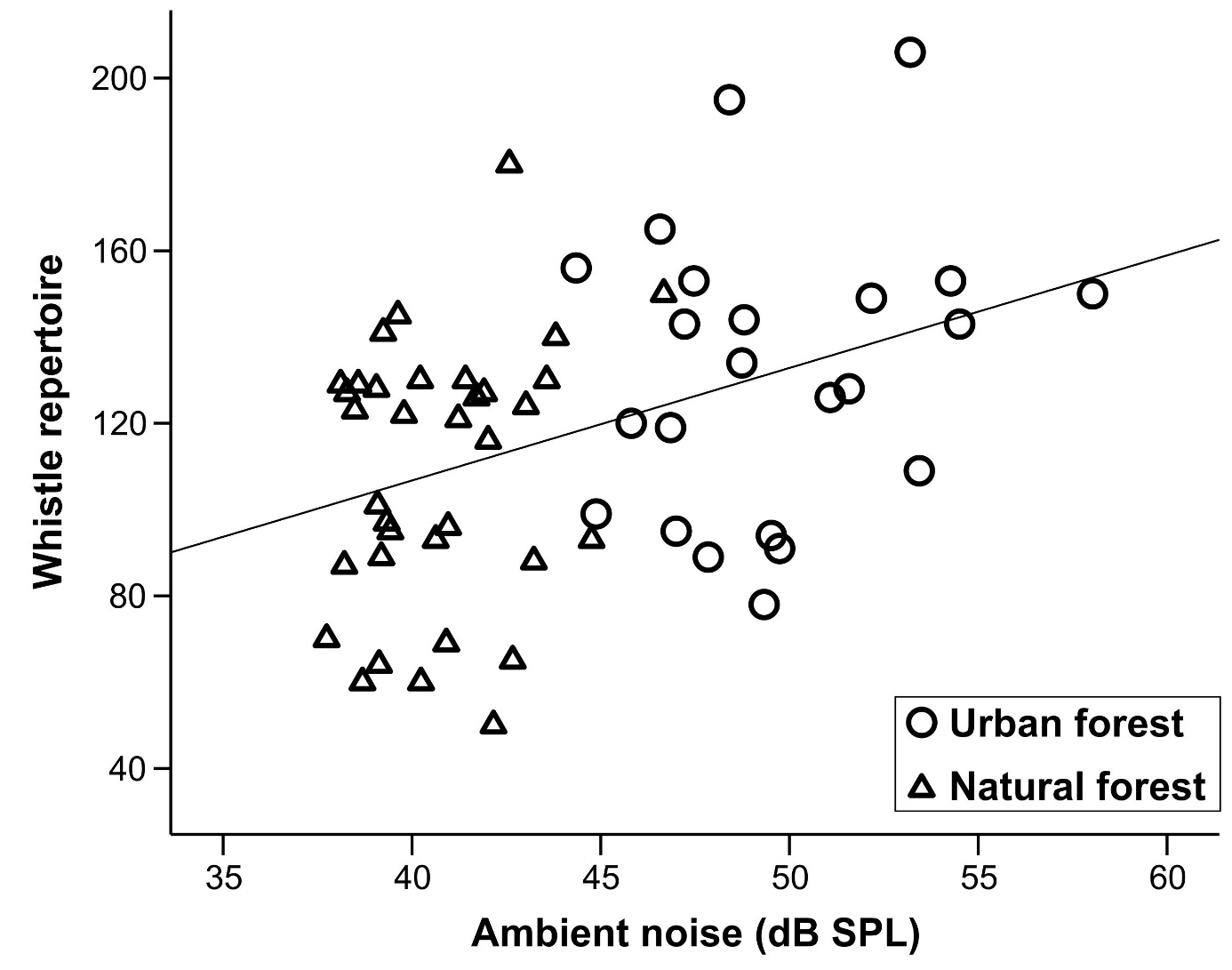
**

**Figure S5:** Relationship between whistle repertoire size (number of unique whistle syllable types within 1000 subsequent syllables of continuous song) and ambient noise level in studied habitats.

**
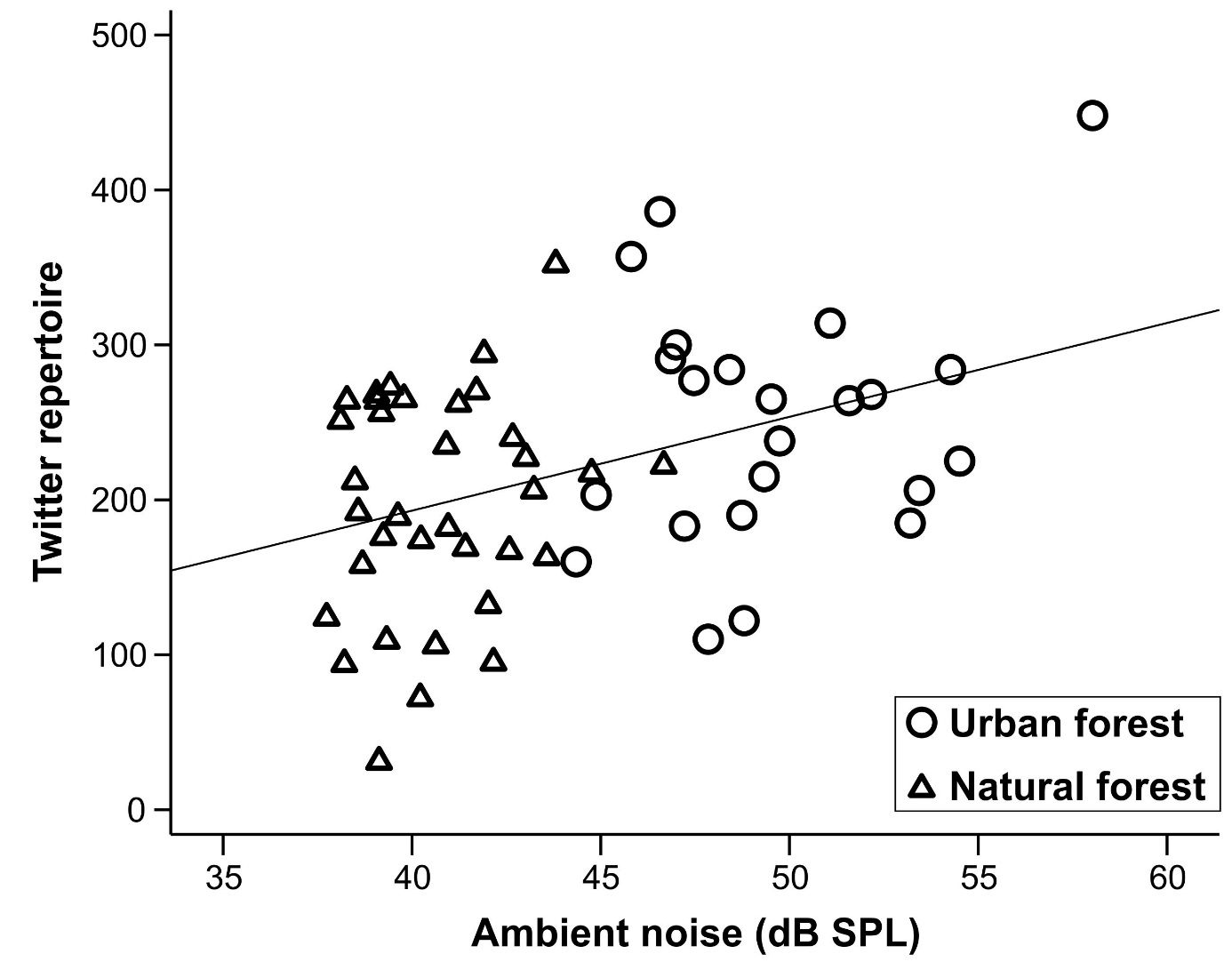
**

**Figure S6:** Relationship between twitter repertoire size (number of unique twitter syllable types within 1000 subsequent syllables of continuous song) and ambient noise level in studied habitats.

**
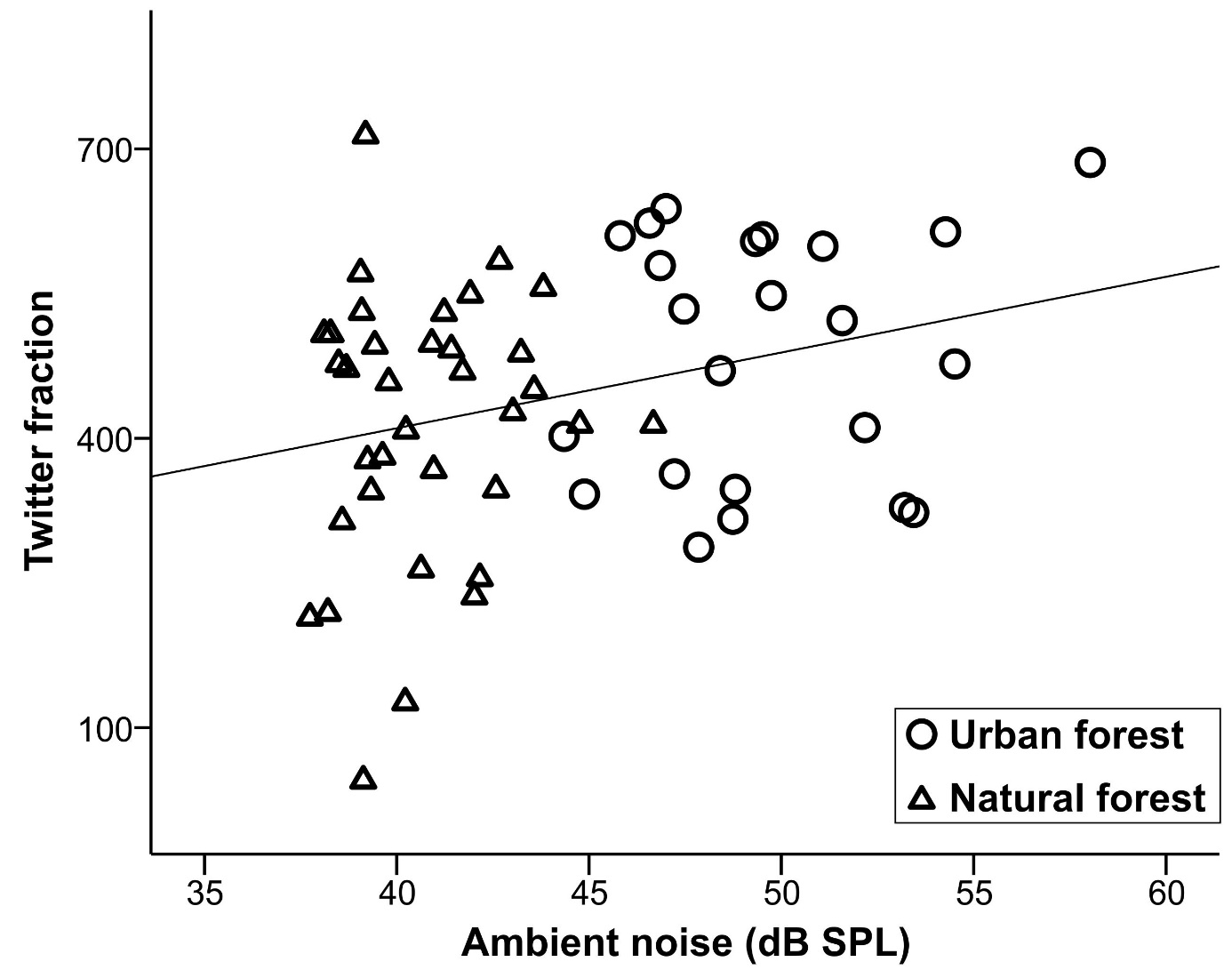
**

**Figure S7:** Relationship between twitter fraction (number of twitter syllables within 1000 subsequent syllables of continuous song) and ambient noise level in studied habitats.


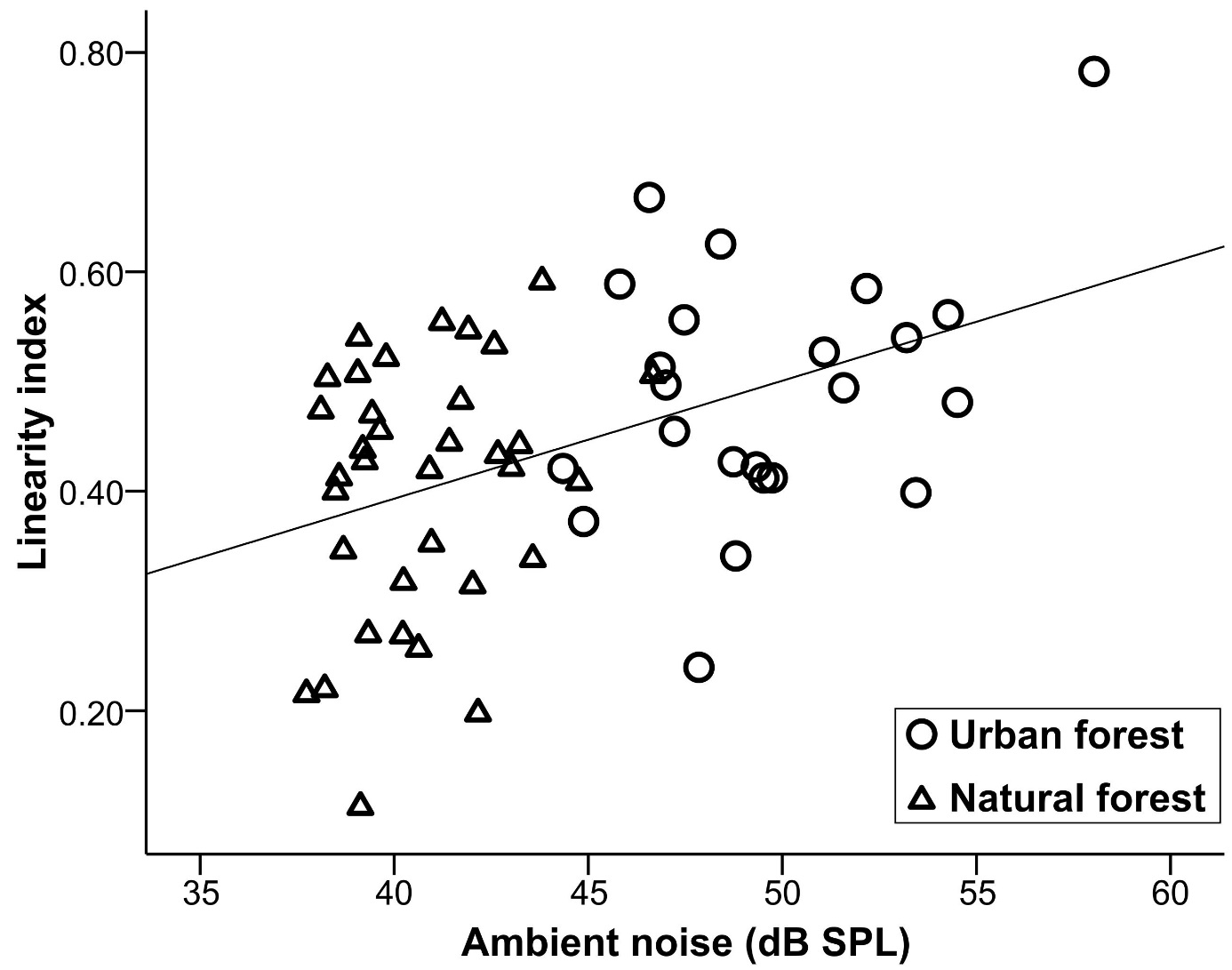


**Figure S8.** Relationship between linearity index and ambient noise level in studied habitats.
