# Supplementary material for: Habitat-related differences in song structure and complexity in a songbird with a large repertoire
Source: BMC Ecol. 2019 Sep 18;19:40. doi: 10.1186/s12898-019-0255-7 (PMC6749692; doi:10.1186/s12898-019-0255-7)
Supplement: Supplementary file 2 — Additional file 2: Table S1. Correlation matrices of song parameters from song thrush males recorded in urban forests and natural forests. Significant values are indicated in bold. Table S2. Correlation matrices of song parameters from song thrush males recorded in urban forests. Significant values are indicated in bold. Table S3. Correlation matrices of song parameters from song thrush males recorded in natural forests. Significant values are indicated in bold. Table S4. Results of the best fitting general linear models explaining the variation in song characteristics that differed between the studied habitats. Table S5. Results of the linear regression models explaining the relationship between ambient noise levels on song thrush song characteristics. Models show results for urban habitat, forest habitat and the data combined. [file 12898_2019_255_MOESM2_ESM.docx]

**Additional file 1: Tables S1-S5**

**BMC Ecology**

**DOI: 10.1186/s12898-019-0255-7**

**Title:** Habitat-related differences in song structure and complexity in a songbird with a large repertoire

**Authors:** Krzysztof Deoniziak ^1, 2^, Tomasz S. Osiejuk ^1^

**Affiliation:**

^1^ Department of Behavioural Ecology, Institute of Environmental Sciences, Faculty of Biology,
Adam Mickiewicz University, Umultowska 89, 61-614 Poznań, Poland

^2^ Laboratory of Insect Evolutionary Biology and Ecology, Institute of Biology, University of Bialystok, Ciołkowskiego 1J, 15-245 Białystok, Poland

**Corresponding author email:** [krzysztofdeo@gmail.com](mailto:krzysztofdeo@gmail.com)

Table S1. Correlation matrices of song parameters from song thrush males recorded in urban forests and natural forests. Significant values are indicated in bold.

|  | Whistle minimum frequency | | Whistle peak frequency | | Twitter minimum frequency | | Twitter peak frequency | | Syllable repertoire | | Whistle repertoire | | Twitter repertoire | | Twitter fraction | | Syllable duration | | Inter-syllable intervals | | Syllable rate | | Redundancy index | | Linearity index | |
| --- | --- | --- | --- | --- | --- | --- | --- | --- | --- | --- | --- | --- | --- | --- | --- | --- | --- | --- | --- | --- | --- | --- | --- | --- | --- | --- |
|  | r | p | r | p | r | p | r | p | r | p | r | p | r | p | r | p | r | p | r | p | r | p | r | p | r | p |
| Whistle minimum frequency | 1.000 |  | 0.836 | **<0.001** | 0.219 | 0.099 | 0.148 | 0.268 | 0.185 | 0.164 | 0.172 | 0.196 | 0.151 | 0.257 | 0.136 | 0.309 | -0.038 | 0.778 | -0.041 | 0.761 | 0.050 | 0.711 | -0.075 | 0.576 | 0.167 | 0.210 |
| Whistle peak frequency | 0.836 | **<0.001** | 1.000 |  | 0.172 | 0.196 | 0.167 | 0.211 | 0.351 | **0.007** | 0.278 | **0.035** | 0.307 | **0.019** | 0.335 | **0.010** | 0.003 | 0.984 | -0.090 | 0.501 | 0.059 | 0.659 | -0.025 | 0.853 | 0.336 | **0.010** |
| Twitter minimum frequency | 0.219 | 0.099 | 0.172 | 0.196 | 1.000 |  | 0.927 | **<0.001** | 0.317 | **0.015** | 0.556 | **<0.001** | 0.148 | 0.267 | 0.092 | 0.493 | -0.133 | 0.319 | -0.078 | 0.559 | 0.133 | 0.319 | 0.089 | 0.505 | 0.339 | **0.009** |
| Twitter peak frequency | 0.148 | 0.268 | 0.167 | 0.211 | 0.927 | **<0.001** | 1.000 |  | 0.373 | **0.004** | 0.495 | **<0.001** | 0.242 | 0.067 | 0.206 | **0.120** | -0.162 | 0.223 | -0.151 | 0.259 | 0.198 | 0.136 | 0.044 | 0.744 | 0.381 | **0.003** |
| Syllable repertoire | 0.185 | 0.164 | 0.351 | **0.007** | 0.317 | **0.015** | 0.373 | **0.004** | 1.000 |  | 0.627 | **<0.001** | 0.944 | **<0.001** | 0.750 | **<0.001** | -0.392 | **0.002** | -0.487 | **<0.001** | 0.496 | **<0.001** | -0.038 | 0.775 | 0.969 | **<0.001** |
| Whistle repertoire | 0.172 | 0.196 | 0.278 | **0.035** | 0.556 | **<0.001** | 0.495 | **<0.001** | 0.627 | **<0.001** | 1.000 |  | 0.336 | **0.010** | 0.125 | 0.349 | -0.076 | 0.573 | -0.138 | 0.303 | 0.128 | 0.339 | 0.054 | 0.686 | 0.638 | **<0.001** |
| Twitter repertoire | 0.151 | 0.257 | 0.307 | **0.019** | 0.148 | 0.267 | 0.242 | 0.067 | 0.944 | **<0.001** | 0.336 | **0.010** | 1.000 |  | 0.855 | **<0.001** | -0.442 | **0.001** | -0.530 | **<0.001** | 0.546 | **<0.001** | -0.069 | 0.605 | 0.902 | **<0.001** |
| Twitter fraction | 0.136 | 0.309 | 0.335 | **0.010** | 0.092 | 0.493 | 0.206 | 0.120 | 0.750 | **<0.001** | 0.125 | 0.349 | 0.855 | **<0.001** | 1.000 |  | -0.494 | **<0.001** | -0.595 | **<0.001** | 0.604 | **<0.001** | 0.065 | 0.629 | 0.737 | **<0.001** |
| Syllable duration | -0.038 | 0.778 | 0.003 | 0.984 | -0.133 | 0.319 | -0.162 | 0.223 | -0.392 | **0.002** | -0.076 | 0.573 | -0.442 | **0.001** | -0.494 | **<0.001** | 1.000 |  | 0.578 | **<0.001** | -0.646 | **<0.001** | -0.381 | **0.003** | -0.460 | **<0.001** |
| Inter-syllable intervals | -0.041 | 0.761 | -0.090 | 0.501 | -0.078 | 0.559 | -0.151 | 0.259 | -0.487 | **<0.001** | -0.138 | 0.303 | -0.530 | **<0.001** | -0.595 | **<0.001** | 0.578 | **<0.001** | 1.000 |  | -0.937 | **<0.001** | -0.194 | 0.145 | -0.518 | **<0.001** |
| Syllable rate | 0.050 | 0.711 | 0.059 | 0.659 | 0.133 | 0.319 | 0.198 | 0.136 | 0.496 | **<0.001** | 0.128 | 0.339 | 0.546 | **<0.001** | 0.604 | **<0.001** | -0.646 | **<0.001** | -0.937 | **<0.001** | 1.000 |  | 0.188 | 0.158 | 0.529 | **<0.001** |
| Redundancy index | -0.075 | 0.576 | -0.025 | 0.853 | 0.089 | 0.505 | 0.044 | 0.744 | -0.038 | 0.775 | 0.054 | 0.686 | -0.069 | 0.605 | 0.065 | **0.629** | -0.381 | **0.003** | -0.194 | 0.145 | 0.188 | 0.158 | 1.000 |  | 0.201 | 0.130 |
| Linearity index | 0.167 | 0.210 | 0.336 | **0.010** | 0.339 | **0.009** | 0.381 | **0.003** | 0.969 | **<0.001** | 0.638 | **<0.001** | 0.902 | **<0.001** | 0.737 | **<0.001** | -0.460 | **<0.001** | -0.518 | **<0.001** | 0.529 | **<0.001** | 0.201 | 0.130 | 1.000 |  |

Table S2. Correlation matrices of song parameters from song thrush males recorded in urban forests. Significant values are indicated in bold.

|  | Whistle minimum frequency | | Whistle peak frequency | | Twitter minimum frequency | | Twitter peak frequency | | Syllable repertoire | | Whistle repertoire | | Twitter repertoire | | Twitter fraction | | Syllable duration | | Inter-syllable intervals | | Syllable rate | | Redundancy index | | Linearity index | |
| --- | --- | --- | --- | --- | --- | --- | --- | --- | --- | --- | --- | --- | --- | --- | --- | --- | --- | --- | --- | --- | --- | --- | --- | --- | --- | --- |
|  | r | p | r | p | r | p | r | p | r | p | r | p | r | p | r | p | r | p | r | p | r | p | r | p | r | p |
| Whistle minimum frequency | 1.000 |  | 0.802 | **<0.001** | 0.225 | 0.301 | 0.248 | 0.253 | -0.048 | 0.828 | 0.060 | 0.787 | -0.079 | 0.720 | 0.065 | 0.769 | 0.148 | 0.501 | 0.126 | 0.566 | -0.112 | 0.612 | 0.031 | 0.887 | -0.056 | 0.801 |
| Whistle peak frequency | 0.802 | **<0.001** | 1.000 |  | 0.274 | 0.206 | 0.340 | 0.112 | 0.028 | 0.898 | 0.101 | 0.647 | -0.009 | 0.967 | 0.146 | 0.505 | 0.231 | 0.290 | 0.069 | 0.755 | -0.109 | 0.620 | 0.068 | 0.759 | 0.022 | 0.920 |
| Twitter minimum frequency | 0.225 | 0.301 | 0.274 | 0.206 | 1.000 |  | 0.926 | **<0.001** | 0.446 | **0.033** | 0.629 | **0.001** | 0.248 | 0.254 | 0.164 | 0.455 | -0.107 | 0.628 | -0.072 | 0.745 | 0.107 | 0.626 | 0.287 | 0.184 | 0.504 | **0.014** |
| Twitter peak frequency | 0.248 | 0.253 | 0.340 | 0.112 | 0.926 | **<0.001** | 1.000 |  | 0.450 | **0.031** | 0.489 | **0.018** | 0.310 | 0.150 | 0.246 | 0.258 | -0.102 | 0.642 | -0.171 | 0.434 | 0.166 | 0.450 | 0.228 | 0.294 | 0.494 | **0.017** |
| Syllable repertoire | -0.048 | 0.828 | 0.028 | 0.898 | 0.446 | **0.033** | 0.450 | **0.031** | 1.000 |  | 0.488 | **0.018** | 0.934 | **<0.001** | 0.675 | **<0.001** | -0.626 | **0.001** | -0.429 | **0.041** | 0.464 | **0.026** | -0.063 | 0.774 | 0.975 | **<0.001** |
| Whistle repertoire | 0.060 | 0.787 | 0.101 | 0.647 | 0.629 | **0.001** | 0.489 | **0.018** | 0.488 | **0.018** | 1.000 |  | 0.143 | 0.516 | -0.143 | 0.516 | -0.121 | 0.582 | 0.264 | 0.224 | -0.189 | 0.387 | 0.362 | 0.089 | 0.565 | **0.005** |
| Twitter repertoire | -0.079 | 0.720 | -0.009 | 0.967 | 0.248 | 0.254 | 0.310 | 0.150 | 0.934 | **<0.001** | 0.143 | 0.516 | 1.000 |  | 0.824 | **<0.001** | -0.660 | **0.001** | -0.595 | **0.003** | 0.604 | **0.002** | -0.221 | 0.312 | 0.874 | **<0.001** |
| Twitter fraction | 0.065 | 0.769 | 0.146 | 0.505 | 0.164 | 0.455 | 0.246 | 0.258 | 0.675 | **<0.001** | -0.143 | 0.516 | 0.824 | **<0.001** | 1.000 |  | -0.451 | **0.031** | -0.651 | **0.001** | 0.605 | **0.002** | -0.257 | 0.236 | 0.603 | **0.002** |
| Syllable duration | 0.148 | 0.501 | 0.231 | 0.290 | -0.107 | 0.628 | -0.102 | 0.642 | -0.626 | **0.001** | -0.121 | 0.582 | -0.660 | **0.001** | -0.451 | **0.031** | 1.000 |  | 0.570 | **0.005** | -0.723 | **<0.001** | 0.085 | 0.701 | -0.603 | **0.002** |
| Inter-syllable intervals | 0.126 | 0.566 | 0.069 | 0.755 | -0.072 | 0.745 | -0.171 | 0.434 | -0.429 | **0.041** | 0.264 | 0.224 | -0.595 | **0.003** | -0.651 | **0.001** | 0.570 | **0.005** | 1.000 |  | -0.954 | **<0.001** | 0.255 | 0.241 | -0.373 | 0.080 |
| Syllable rate | -0.112 | 0.612 | -0.109 | 0.620 | 0.107 | 0.626 | 0.166 | 0.450 | 0.464 | **0.026** | -0.189 | 0.387 | 0.604 | **0.002** | 0.605 | **0.002** | -0.723 | **<0.001** | -0.954 | **<0.001** | 1.000 |  | -0.258 | 0.234 | 0.409 | 0.053 |
| Redundancy index | 0.031 | 0.887 | 0.068 | 0.759 | 0.287 | 0.184 | 0.228 | 0.294 | -0.063 | 0.774 | 0.362 | 0.089 | -0.221 | 0.312 | -0.257 | 0.236 | 0.085 | 0.701 | 0.255 | 0.241 | -0.258 | 0.234 | 1.000 |  | 0.154 | 0.482 |
| Linearity index | -0.056 | 0.801 | 0.022 | 0.920 | 0.504 | **0.014** | 0.494 | **0.017** | 0.975 | **<0.001** | 0.565 | **0.005** | 0.874 | **<0.001** | 0.603 | **0.002** | -0.603 | **0.002** | -0.373 | 0.080 | 0.409 | 0.053 | 0.154 | 0.482 | 1.000 |  |

Table S3. Correlation matrices of song parameters from song thrush males recorded in natural forests. Significant values are indicated in bold.

|  | Whistle minimum frequency | | Whistle peak frequency | | Twitter minimum frequency | | Twitter peak frequency | | Syllable repertoire | | Whistle repertoire | | Twitter repertoire | | Twitter fraction | | Syllable duration | | Inter-syllable intervals | | Syllable rate | | Redundancy index | | Linearity index | |
| --- | --- | --- | --- | --- | --- | --- | --- | --- | --- | --- | --- | --- | --- | --- | --- | --- | --- | --- | --- | --- | --- | --- | --- | --- | --- | --- |
|  | r | p | r | p | r | p | r | p | r | p | r | p | r | p | r | p | r | p | r | p | r | p | r | p | r | p |
| Whistle minimum frequency | 1.000 |  | 0.804 | **<0.001** | 0.103 | 0.555 | -0.019 | 0.916 | 0.030 | 0.862 | 0.001 | 0.996 | 0.037 | 0.835 | 0.005 | 0.977 | -0.073 | 0.679 | -0.013 | 0.941 | 0.033 | 0.851 | -0.019 | 0.915 | 0.037 | 0.835 |
| Whistle peak frequency | 0.804 | **<0.001** | 1.000 |  | -0.005 | 0.975 | -0.036 | 0.837 | 0.329 | 0.053 | 0.191 | 0.273 | 0.317 | 0.063 | 0.310 | 0.070 | -0.058 | 0.741 | -0.081 | 0.643 | 0.064 | 0.715 | 0.032 | 0.855 | 0.326 | 0.056 |
| Twitter minimum frequency | 0.103 | 0.555 | -0.005 | 0.975 | 1.000 |  | 0.924 | **<0.001** | 0.111 | 0.526 | 0.443 | **0.008** | -0.055 | 0.753 | -0.048 | 0.782 | -0.140 | 0.423 | -0.046 | 0.794 | 0.113 | 0.520 | 0.055 | 0.752 | 0.129 | 0.461 |
| Twitter peak frequency | -0.019 | 0.916 | -0.036 | 0.837 | 0.924 | **<0.001** | 1.000 |  | 0.230 | 0.183 | 0.436 | **0.009** | 0.092 | 0.597 | 0.105 | 0.547 | -0.185 | 0.288 | -0.111 | 0.524 | 0.184 | 0.291 | 0.019 | 0.914 | 0.228 | 0.187 |
| Syllable repertoire | 0.030 | 0.862 | 0.329 | 0.053 | 0.111 | 0.526 | 0.230 | 0.183 | 1.000 |  | 0.628 | **<0.001** | 0.943 | **<0.001** | 0.766 | **<0.001** | -0.293 | 0.087 | -0.521 | **0.001** | 0.512 | **0.002** | 0.065 | 0.712 | 0.959 | **<0.001** |
| Whistle repertoire | 0.001 | 0.996 | 0.191 | 0.273 | 0.443 | **0.008** | 0.436 | **0.009** | 0.628 | **<0.001** | 1.000 |  | 0.333 | 0.051 | 0.152 | 0.382 | -0.033 | 0.852 | -0.277 | 0.107 | 0.270 | 0.116 | 0.002 | 0.993 | 0.604 | **<0.001** |
| Twitter repertoire | 0.037 | 0.835 | 0.317 | 0.063 | -0.055 | 0.753 | 0.092 | 0.597 | 0.943 | **<0.001** | 0.333 | 0.051 | 1.000 |  | 0.863 | **<0.001** | -0.341 | **0.045** | -0.512 | **0.002** | 0.505 | **0.002** | 0.078 | 0.657 | 0.903 | **<0.001** |
| Twitter fraction | 0.005 | 0.977 | 0.310 | 0.070 | -0.048 | 0.782 | 0.105 | 0.547 | 0.766 | **<0.001** | 0.152 | 0.382 | 0.863 | **<0.001** | 1.000 |  | -0.526 | **0.001** | -0.572 | **<0.001** | 0.595 | **<0.001** | 0.258 | 0.135 | 0.781 | **<0.001** |
| Syllable duration | -0.073 | 0.679 | -0.058 | 0.741 | -0.140 | 0.423 | -0.185 | 0.288 | -0.293 | 0.087 | -0.033 | 0.852 | -0.341 | **0.045** | -0.526 | **0.001** | 1.000 |  | 0.582 | **<0.001** | -0.612 | **<0.001** | -0.563 | **<0.001** | -0.412 | **0.014** |
| Inter-syllable intervals | -0.013 | 0.941 | -0.081 | 0.643 | -0.046 | 0.794 | -0.111 | 0.524 | -0.521 | **0.001** | -0.277 | 0.107 | -0.512 | **0.002** | -0.572 | **<0.001** | 0.582 | **<0.001** | 1.000 |  | -0.941 | **<0.001** | -0.355 | **0.037** | -0.584 | **<0.001** |
| Syllable rate | 0.033 | 0.851 | 0.064 | 0.715 | 0.113 | 0.520 | 0.184 | 0.291 | 0.512 | **0.002** | 0.270 | 0.116 | 0.505 | **0.002** | 0.595 | **<0.001** | -0.612 | **<0.001** | -0.941 | **<0.001** | 1.000 |  | 0.396 | **0.019** | 0.592 | **<0.001** |
| Redundancy index | -0.019 | 0.915 | 0.032 | 0.855 | 0.055 | 0.752 | 0.019 | 0.914 | 0.065 | 0.712 | 0.002 | 0.993 | 0.078 | 0.657 | 0.258 | 0.135 | -0.563 | **<0.001** | -0.355 | **0.037** | 0.396 | **0.019** | 1.000 |  | 0.334 | 0.050 |
| Linearity index | 0.037 | 0.835 | 0.326 | 0.056 | 0.129 | 0.461 | 0.228 | 0.187 | 0.959 | **<0.001** | 0.604 | **<0.001** | 0.903 | **<0.001** | 0.781 | **<0.001** | -0.412 | **0.014** | -0.584 | **<0.001** | 0.592 | **<0.001** | 0.334 | 0.050 | 1.000 |  |

**Table S4.** Results of the best fitting general linear models explaining the variation in song characteristics that differed between the studied habitats.

| Model | Predictors | Estimate | SE | P |
| --- | --- | --- | --- | --- |
| Whistle minimum frequency |  |  |  |  |
| 1 | Intercept | 2366.845 | 22.983 | < 0.001 |
|  | HABITAT | 140.315 | 36.497 | **< 0.001** |
| 2 | Intercept | 2394.357 | 27.751 | < 0.001 |
|  | HABITAT | 140.177 | 36.237 | **< 0.001** |
|  | MALES | -37.035 | 40.483 | 0.360 |
| 3 | Intercept | 2324.045 | 53.187 | < 0.001 |
|  | HABITAT | 143.149 | 36.390 | **< 0.001** |
|  | HOUR | 17.023 | 19.107 | 0.373 |
| Whistle peak frequency |  |  |  |  |
| 1 | Intercept | 2874.77 | 31.320 | < 0.001 |
|  | HABITAT | 170.506 | 49.736 | **0.001** |
| 2 | Intercept | 2801.209 | 72.186 | < 0.001 |
|  | HABITAT | 175.376 | 49.388 | **< 0.001** |
|  | HOUR | 29.257 | 25.932 | 0.259 |
| 3 | Intercept | 2933.751 | 66.635 | < 0.001 |
|  | HABITAT | 156.276 | 51.323 | **0.002** |
|  | DAY | -1.155 | 1.155 | 0.317 |
| 4 | Intercept | 2859.892 | 85.526 | < 0.001 |
|  | HABITAT | 158.679 | 50.571 | **0.002** |
|  | DAY | -1.433 | 1.156 | 0.215 |
|  | HOUR | 35.025 | 26.014 | 0.178 |
| 5 | Intercept | 3104.088 | 369.822 | < 0.001 |
|  | HABITAT | 220.419 | 94.290 | **0.019** |
|  | NOISE | -5.630 | 9.046 | 0.534 |
| Syllable repertoire |  |  |  |  |
| 1 | Intercept | 42.043 | 108.324 | 0.698 |
|  | DAY | -0.869 | 0.520 | 0.095 |
|  | NOISE | 7.562 | 2.218 | **0.001** |
| 2 | Intercept | -46.920 | 96.565 | 0.627 |
|  | NOISE | 8.666 | 2.168 | **< 0.001** |
| 3 | Intercept | -11.002 | 118.215 | 0.926 |
|  | DAY | -0.937 | 2.258 | 0.071 |
|  | HOUR | 12.848 | 12.030 | 0.286 |
|  | NOISE | 8.121 | 2.258 | **< 0.001** |
| 4 | Intercept | 56.559 | 109.324 | 0.605 |
|  | DAY | -0.832 | 0.520 | 0.109 |
|  | MALES | -19.381 | 24.664 | 0.432 |
|  | NOISE | 7.520 | 2.207 | **0.001** |
| 5 | Intercept | -25.222 | 98.765 | 0.798 |
|  | MALES | -22.965 | 25.099 | 0.360 |
|  | NOISE | 8.560 | 2.156 | **< 0.001** |
| Whistle repertoire |  |  |  |  |
| 1 | Intercept | 2.394 | 24.919 | 0.945 |
|  | NOISE | 2.609 | 0.784 | **0.001** |
| 2 | Intercept | 14.792 | 35.326 | 0.675 |
|  | MALES | -13.122 | 8.977 | 0.144 |
|  | NOISE | 2.548 | 0.771 | **0.001** |
| 3 | Intercept | -16.127 | 40.398 | 0.690 |
|  | HOUR | 3.962 | 4.432 | 0.371 |
|  | NOISE | 2.808 | 0.810 | **0.001** |
| 4 | Intercept | -26.607 | 43.749 | 0.543 |
|  | DAY | 0.118 | 0.192 | 0.540 |
|  | HOUR | 3.625 | 4.452 | 0.415 |
|  | NOISE | 2.941 | 0.8358 | **< 0.001** |
| 5 | Intercept | -11.639 | 39.926 | 0.771 |
|  | DAY | 0.137 | 0.192 | 0.475 |
|  | NOISE | 2.783 | 0.818 | **0.001** |
| Twitter repertoire |  |  |  |  |
| 1 | Intercept | 53.682 | 91.170 | 0.556 |
|  | DAY | -1.006 | 0.438 | **0.022** |
|  | NOISE | 4.779 | 1.867 | **0.010** |
| 2 | Intercept | 15.605 | 99.760 | 0.876 |
|  | DAY | -1.055 | 0.438 | **0.016** |
|  | HOUR | 9.222 | 10.152 | 0.364 |
|  | NOISE | 5.181 | 1.906 | **0.007** |
| Twitter fraction |  |  |  |  |
| 1 | Intercept | 232.520 | 169.912 | 0.171 |
|  | DAY | -1.330 | 0.816 | 0.103 |
|  | NOISE | 6.161 | 3.480 | 0.077 |
| 2 | Intercept | 96.327 | 151.302 | 0.524 |
|  | NOISE | 7.851 | 3.397 | **0.021** |
| 3 | Intercept | 486.226 | 47.049 | <0.001 |
|  | DAY | -1.408 | 0.815 | 0.118 |
|  | HABITAT | 56.609 | 36.238 | 0.084 |
| 4 | Intercept | 525.013 | 40.812 | <0.001 |
|  | DAY | -1.761 | 0.7997 | **0.028** |
| 5 | Intercept | 414.400 | 22.482 | <0.001 |
|  | HABITAT | 73.948 | 35.702 | **0.038** |
| Linearity index |  |  |  |  |
| 1 | Intercept | -0.037 | 0.126 | 0.770 |
|  | NOISE | 0.011 | 0.003 | **<0.001** |
| 2 | Intercept | 0.036 | 0.143 | 0.803 |
|  | DAY | -0.001 | 0.001 | 0.303 |
|  | NOISE | 0.010 | 0.003 | **0.001** |
| 3 | Intercept | -0.094 | 0.146 | 0.520 |
|  | HOUR | 0.012 | 0.016 | 0.446 |
|  | NOISE | 0.011 | 0.003 | **<0.001** |
| 4 | Intercept | -0.014 | 0.129 | 0.912 |
|  | MALES | -0.024 | 0.033 | 0.468 |
|  | NOISE | 0.011 | 0.003 | **<0.001** |

Results of best fitting general linear models (Δ AIC_C_ < 2) are shown. Predictor codes: DAY, day of season; HOUR, hour after sunrise; NOISE, background noise level; HABITAT, habitat type; MALES, other singing males in hearing range during recording. Significant values are indicated in bold.

**Table S5**. Results of the linear regression models explaining the relationship between ambient noise levels on song thrush song characteristics. Models show results for urban habitat, forest habitat and the data combined.

| Model | Predictors | Estimate | SE | P |
| --- | --- | --- | --- | --- |
| Whistle low frequency |  |  |  |  |
| Urban | Intercept | 2965.789 | 287.023 | < 0.001 |
|  | NOISE | -9.246 | 5.773 | 0.124 |
| Forest | Intercept | 1560.681 | 511.307 | 0.004 |
|  | NOISE | 19.791 | 12.535 | 0.124 |
| Combined | Intercept | 1896.344 | 163.123 | < 0.001 |
|  | NOISE | 11.890 | 3.662 | 0.002 |
| Whistle peak frequency |  |  |  |  |
| Urban | Intercept | 3766,713 | 463.512 | < 0.001 |
|  | NOISE | -14.545 | 9.323 | 0.134 |
| Forest | Intercept | 2493.801 | 684.644 | 0.001 |
|  | NOISE | 9.352 | 16.785 | 0.581 |
| Combined | Intercept | 2395.463 | 225.523 | < 0.001 |
|  | NOISE | 12.360 | 5.063 | 0.018 |
| Syllable repertoire |  |  |  |  |
| Urban | Intercept | -18.846 | 262.590 | 0.943 |
|  | NOISE | 8.106 | 5.528 | 0.125 |
| Forest | Intercept | -69.423 | 272.703 | 0.799 |
|  | NOISE | 9.214 | 6.686 | 0.168 |
| Combined | Intercept | -46.920 | 96.565 | 0.627 |
|  | NOISE | 8.666 | 2.168 | < 0.001 |
| Whistle repertoire |  |  |  |  |
| Urban | Intercept | 28.491 | 97.465 | 0.770 |
|  | NOISE | 2.089 | 1.961 | 0.287 |
| Forest | Intercept | -14.900 | 96.640 | 0.877 |
|  | NOISE | 3.028 | 2.369 | 0.201 |
| Combined | Intercept | 2.394 | 34.919 | 0.945 |
|  | NOISE | 2.609 | 0.784 | 0.001 |
| Twitter repertoire |  |  |  |  |
| Urban | Intercept | -47.336 | 234.996 | 0.840 |
|  | NOISE | 6.016 | 4.727 | 0.203 |
| Forest | Intercept | -54.523 | 227.173 | 0.810 |
|  | NOISE | 6.186 | 5.569 | 0.267 |
| Combined | Intercept | -49.314 | 82.920 | 0.552 |
|  | NOISE | 6.057 | 1.862 | 0.001 |
| Twitter fraction |  |  |  |  |
| Urban | Intercept | 211.667 | 380.244 | 0.578 |
|  | NOISE | 5.578 | 7.648 | 0.466 |
| Forest | Intercept | 79.008 | 447.804 | 0.860 |
|  | NOISE | 8.234 | 10.979 | 0.453 |
| Combined | Intercept | 96.327 | 151.302 | 0.524 |
|  | NOISE | 7.851 | 3.397 | 0.021 |
| Linearity index |  |  |  |  |
| Urban | Intercept | -0.120 | 0.329 | 0.716 |
|  | NOISE | 0.012 | 0.007 | 0.062 |
| Forest | Intercept | -0.155 | 0.364 | 0.671 |
|  | NOISE | 0.014 | 0.009 | 0.125 |
| Combined | Intercept | -0.37 | 0.126 | 0.770 |
|  | NOISE | 0.011 | 0.003 | < 0.001 |
